# Supplementary material for: Bipolar cell targeted optogenetic gene therapy restores parallel retinal signaling and high-level vision in the degenerated retina
Source: Commun Biol. 2022 Oct 20;5:1116. doi: 10.1038/s42003-022-04016-1 (PMC9585040; doi:10.1038/s42003-022-04016-1)
Supplement: Supplementary file 2 — Supplementary Information [file 42003_2022_4016_MOESM2_ESM.pdf]

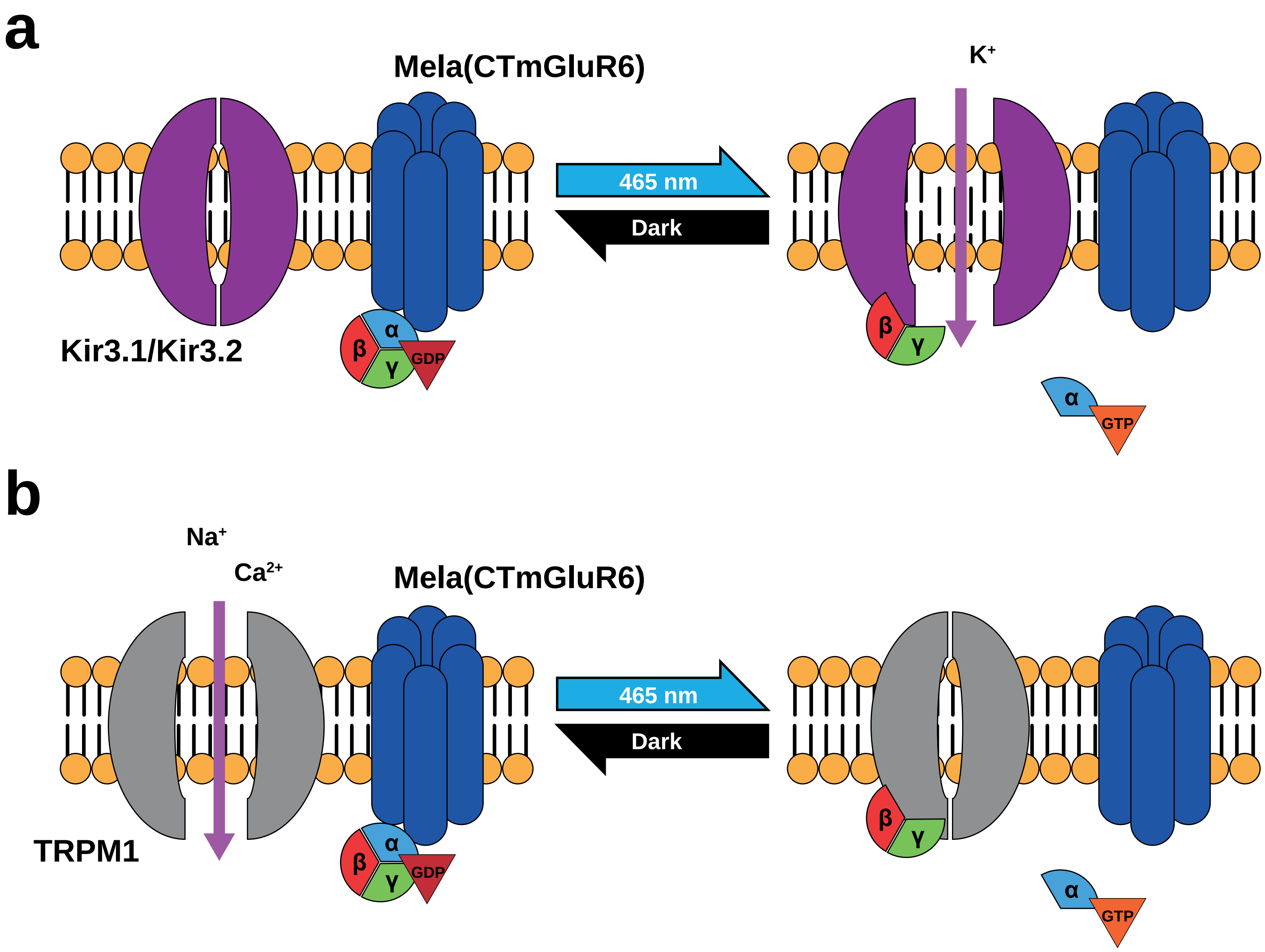

Supplementary Fig. 1: Depiction of mechanisms of action of Mela(CTmGluR6) in HEK-293 GIRK cells and OBCs of the mouse retina. (**a**) HEK293-GIRK cells stably express the Kir3.1/Kir3.2 pot assium channel. Light-activated Mela(CTmGluR6) activates an endogenous Gi/o protein, of which the dissociated Gβγ subunits trigger opening of the GIRK channels leading to potassium influx. (**b**) The non-selective TRPM1 cation channels located in the OBCs of the retina are also Gβγ-gated. Light activation of Mela(CTmGluR6) in this scenario leads to closure of TRPM1 channels and subsequent hyperpolarization of the cell. This is in direct opposition to the action that takes place in photoreceptor gated signal transmission in the healthy retina, where mGluR6 is stimulated by glutamate released from photoreceptors in the dark and TRPM1 channels are opened during light stimulation due to a reduction in glutamate release (see Fig. S9).

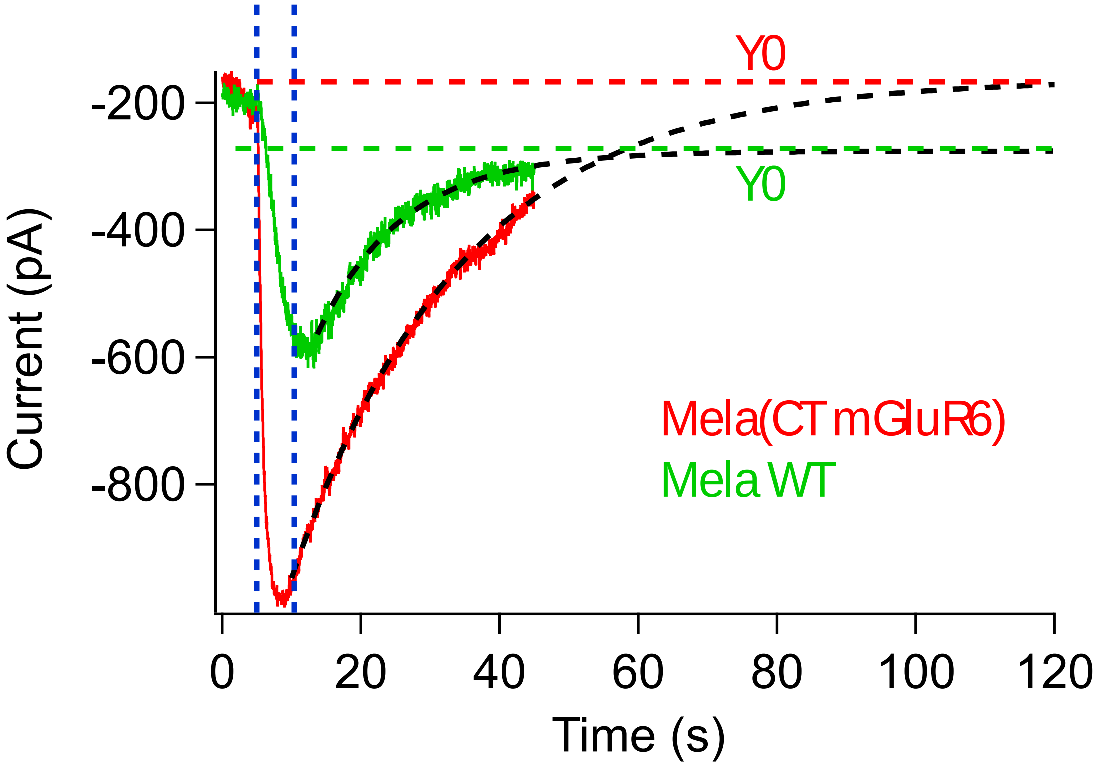

Supplementary Fig. 2: Inactivation of GIRK currents triggered by wild-type melanopsin was incomplete. An exponential fit to recovering GIRK currents predicts an exponential decay to baseline for Mela(CT mGluR6) but an incomplete decay for Mela WT. This suggests that wild-type melanopsin has a double exponential decay with a relatively slow second component that is not present in Mela(CT mGluR6). We were not able to resolve this slow component in our patch-clamp recordings on HEK293-GIRK cells.

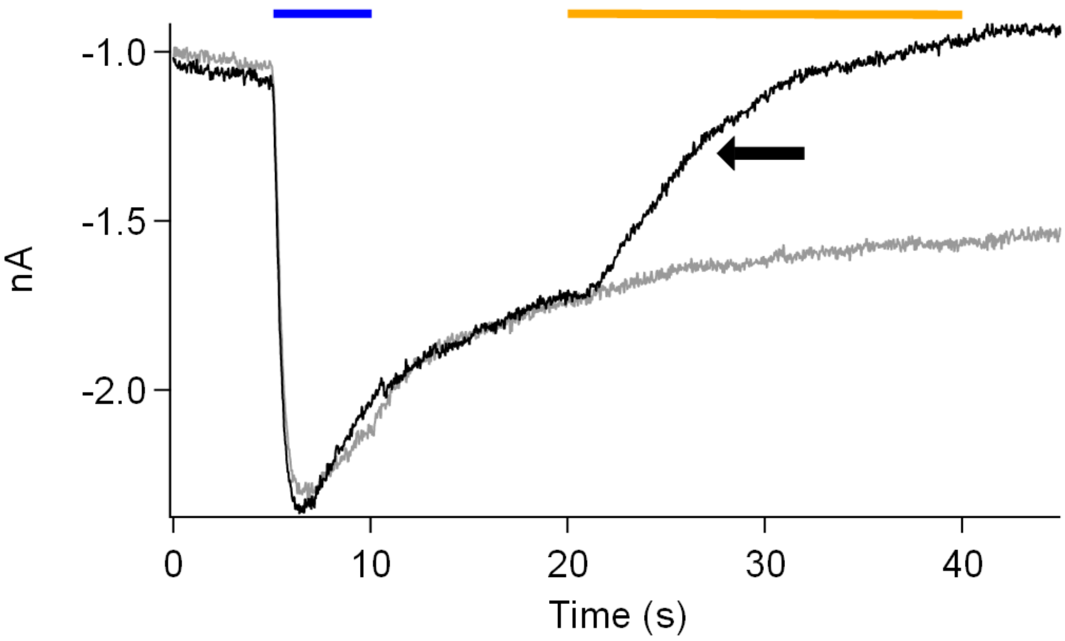

Supplementary Fig. 3: Recovering photoactivatable Mela(CTmGluR6) by orange light. Two light responses recorded from the same HEK-GIRK cell to a blue light stimulus (475nm; 5 x 10^{13} photons/sec/cm^2) with (black trace) and without (grey trace) a subsequent orange light stimulus (595nm; 5 x 10^{15} photons/sec/cm^2) clearly demonstrates the bi-stability of Mela(CTmGluR6).

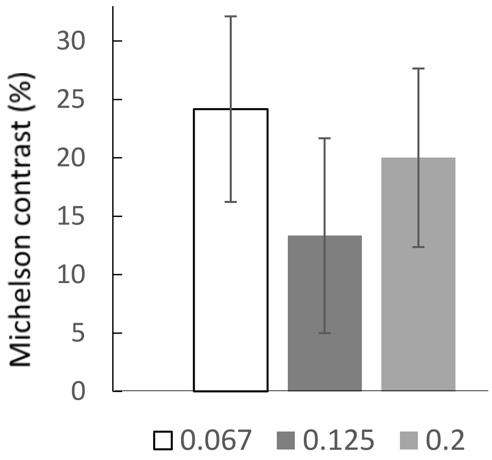

Supplementary Fig. 4: Dependency of the restored contrast resolution on the spatial grating frequencies in treated *rd1* mice. *rd1* mice were treated with an ON-bipolar cell targeted Mela(CTmGluR6) gene therapy. Three treated *rd1* mice were tested on three consecutive days at the three indicated spatial frequencies (0.067, 0.125, 0.2 cyc/deg). The rotation speed of the optokinetic drum was fixed to 12°/sec. Measured was Michelson contrast, defined as (Lmax-Lmin)/(Lmax+Lmin), with Lmax and Lmin representing the highest and lowest luminance, respectively. A spatial frequency of 0.125 cyc/deg resulted in best contrast resolution, although differences were not significant ($p \geq 0.4$; Student's t-test). The spatial frequency setting was therefore in all contrast measurements fixed to 0.125 cycles per degree. Shown are means ± SD.

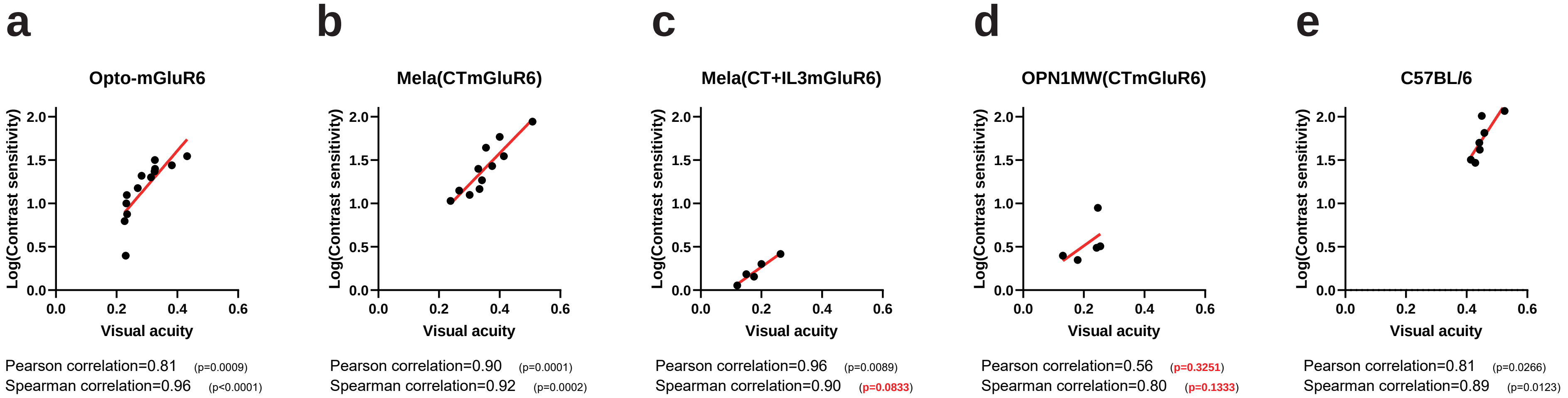

Supplementary Fig. 5: Correlation of visual acuity and contrast sensitivity measured during OMR task. Mice treated with melanopsin-based opotgenetic tools (**a**, n=13; **b**, n=11; **c**, n=5) as well as healthy C57BL/6 animals (**e**, n=7) showed significant positive correlation using Pearson correlation coeficient (α=0.05). Using Spearman correlation coefficient, however, the Mela(CT+IL3mGluR6) mice (**c**) did not reach significant values. The OPN1MW(CTmGluR6) animals (**d**, n=5) did not exhibit significant positive correlations, probably be due to limited sample size. Black dots represent values for individual animals, red lines show simple linear regression. Correlation values and p-values are denoted under the plots.

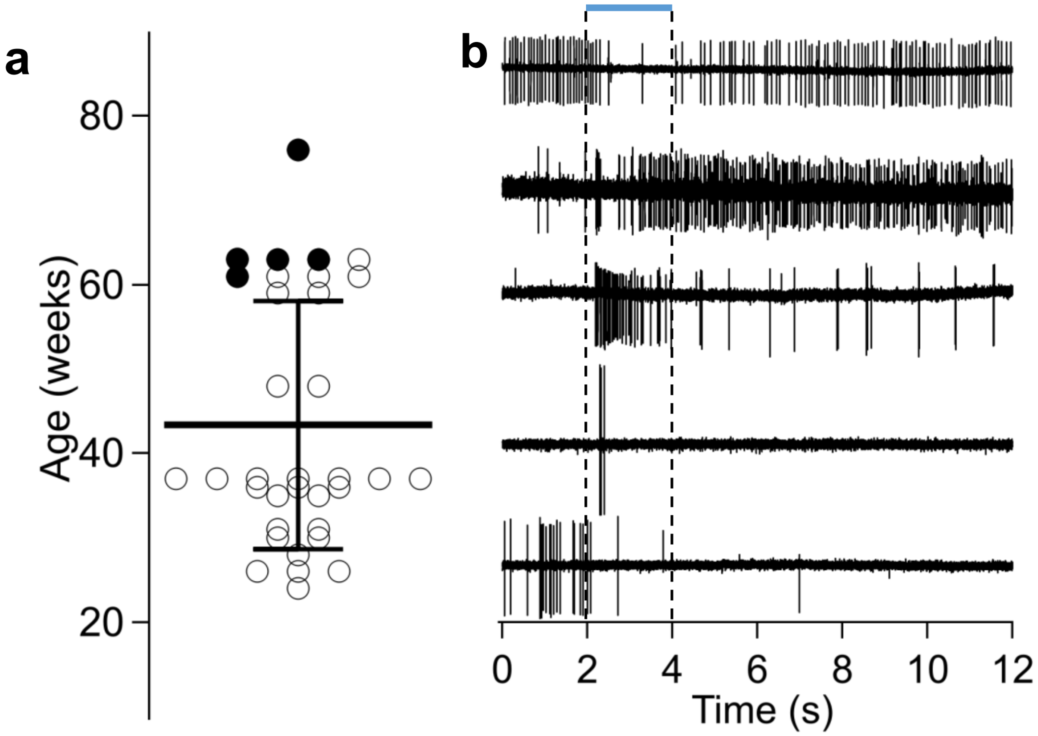

Supplementary Fig. 6: Gene therapy in *rd1* mice remains effective long after photoreceptor degeneration. (**a**) A scatter dot plot showing the age (in weeks) of all rd1 mice used for electrophysiological recording. Ages ranged from 24 to 76 weeks with an average age of 43.4 weeks. Data shown as means ± SD. (**b**) Example extracellular light responses recorded from five of the oldest mice in **a** (filled circles). Older mice had robust light responses with an equal variety of light response types and no apparent differences were observed between different ages. Treated mice were injected a minimum of three weeks prior to experiments but also showed reporter gene expression and light responses up to 42 weeks after treatment, the longest treatment interval tested.

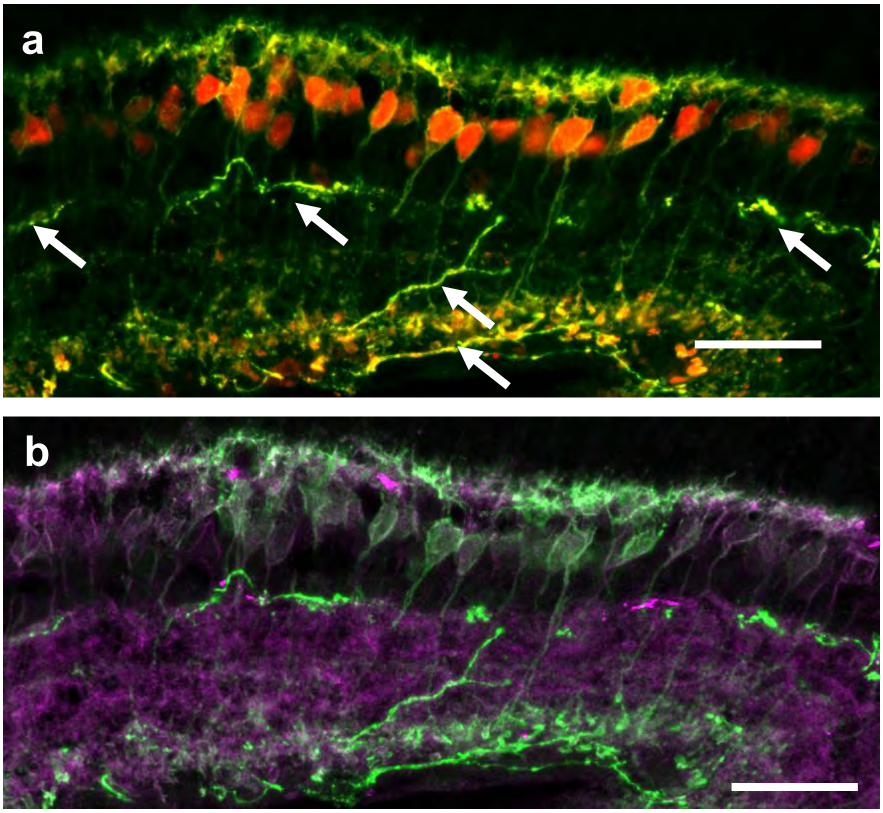

Supplementary Fig. 7: Subcellular targeting of mMela(CTmGluR6) in OBCs. (**a**&**b**) Using the mouse version of Mela-mGluR6 (CT), allowed staining against the N-terminus of mouse melanopsin (rabbit anti-melanopsin; Advanced Targeting Systems; AB-N39). We observed clear melanopsin staining (green) only in transfected, TurboFP635 positive cells (**a**; red), which are also positive for Gαo (**b**; magenta). Arrows in A indicate the processes of ipRGCs. Scale bar = 50 µm.

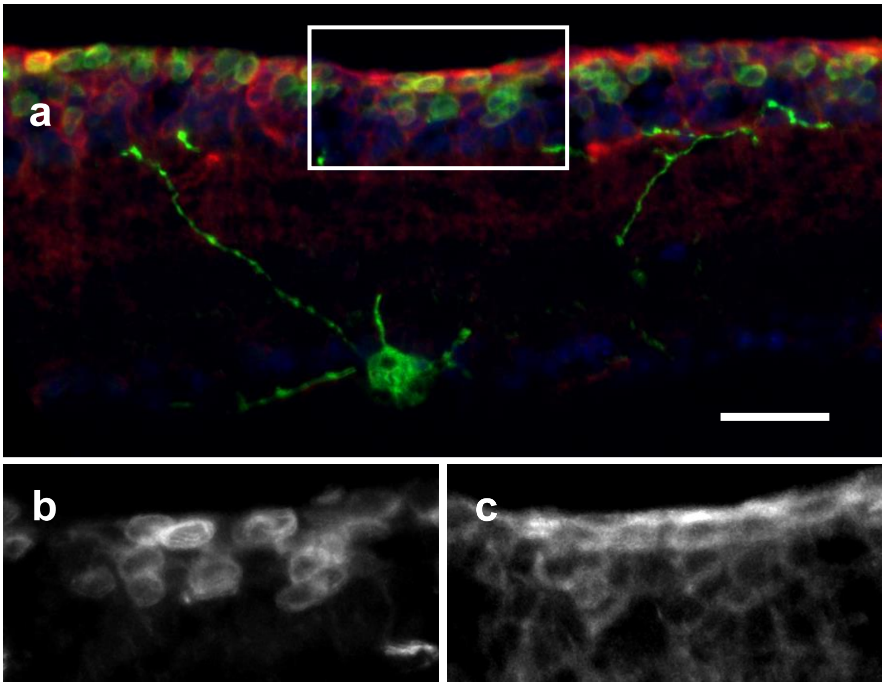

Supplementary Fig. 8: Subcellular targeting of mMela(CTmGluR6) in relation to TRPM1. (**a**) mMela(CTmGluR6) staining (green) largely colocalised with the TRPM1 signal (red) in degenerated *rd1* retinas, where TRPM1 is known to target predominantly to the membrane of the OBC cell bodies. (**b&c**) Higher magnification images of the mMela(CTmGluR6) (green; **b**) and TRPM1 (red; **c**) channels from the insert in panel **a**. Scale bar = 50 µm.

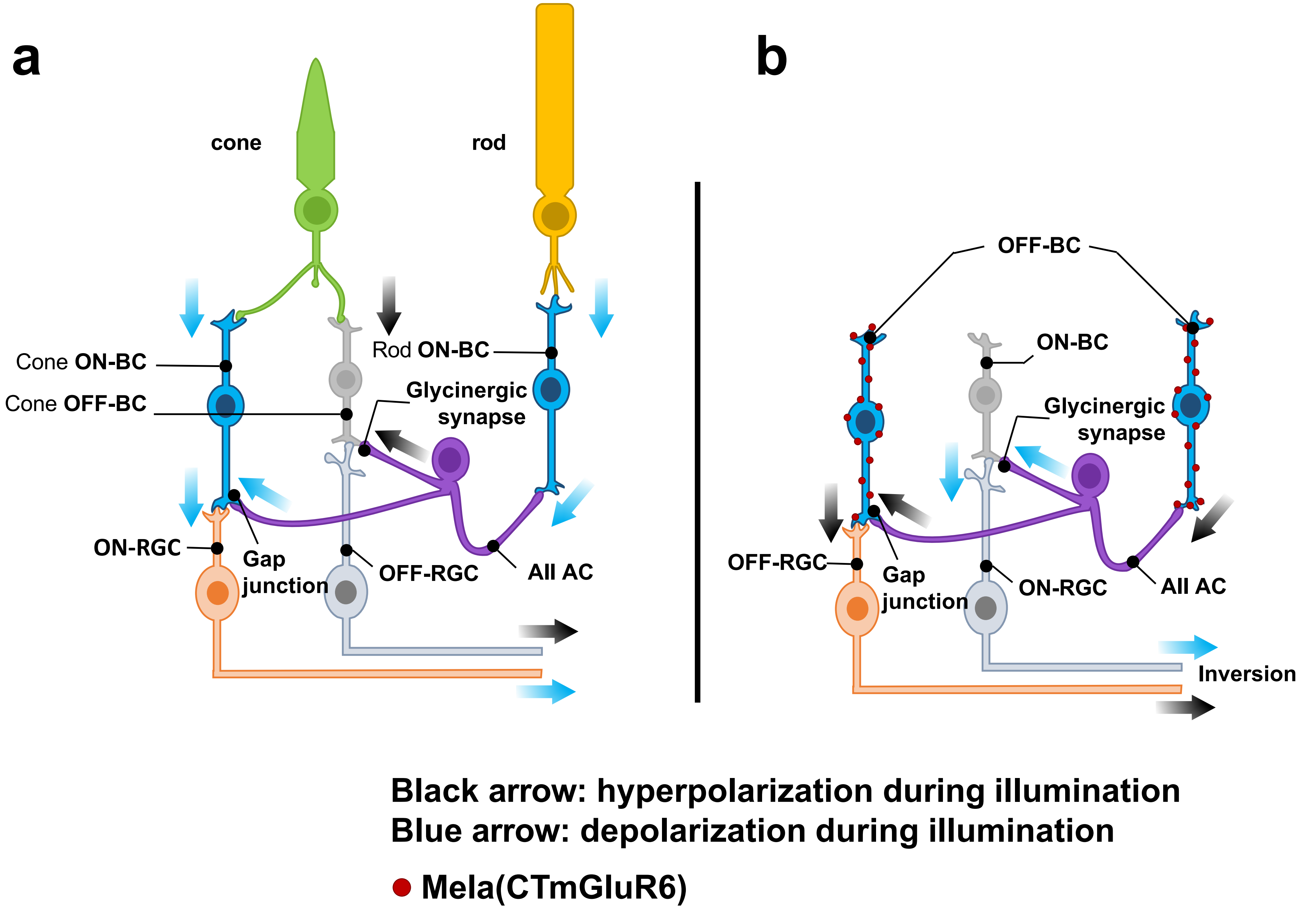

Supplementary Fig. 9: Illustration of the sign inversion of the light signal when OBCs are activated through opsin-mGluR6 chimeras. There are two classes of bipolar cells in the retina, ON- and OFF-bipolar cells, which provide parallel information channels responding to increases and decreases in light intensity, respectively. Whilst cone bipolar cells (receiving input from cones) are divided into ON and OFF type (Cone ON-BC and Cone OFF-BC), rod bipolar cells, receiving input from rods, are all of the ON type (Rod ON-BC). Since rod bipolar cells are by far the most numerous ON-bipolar cells in mice, the majority of opsin-mGluR6 expressing cells will be rod bipolar cells. (**a**) illustrates the intact rod and cone pathways: upon illumination, the rod bipolar cell is depolarized, which subsequently depolarizes the AII amacrine cell (AII AC, magenta). In turn the cone ON-bipolar cell is also depolarized through a sign-conserving electrical synapse, while the cone OFF-bipolar cell is hyperpolarized through a sign-inverting glycinergic synapse. Finally, ON ganglion cells (ON-RGC, orange) are depolarized and OFF ganglion cells (OFF-RGC, grey) are hyperpolarized by light. (**b**) illustrates the degenerated pathway recovered through opsin-mGluR6 (red dots) expressed in ON-bipolar cells. Given the nature of opsin-mGluR6, the original ON-BCs become the new OFF-BCs, and the original OFF-BCs become ON-BCs. Upon illumination, rod bipolar cells and subsequently AII amacrine cells are hyperpolarized. The cone ON-bipolar (now OFF-BCs) cells are also hyperpolarized, directly by light and indirectly by the AII amacrine cells, and the cone OFF-bipolar (now ON-BCs) cells are depolarized through the sign-inverting glycinergic synapse from AII cells. Therefore, native ON-RGCs (orange) are now hyperpolarized (now OFF-RGCs), and native OFF-RGCs (grey) are now depolarized (now ON-RGCs).

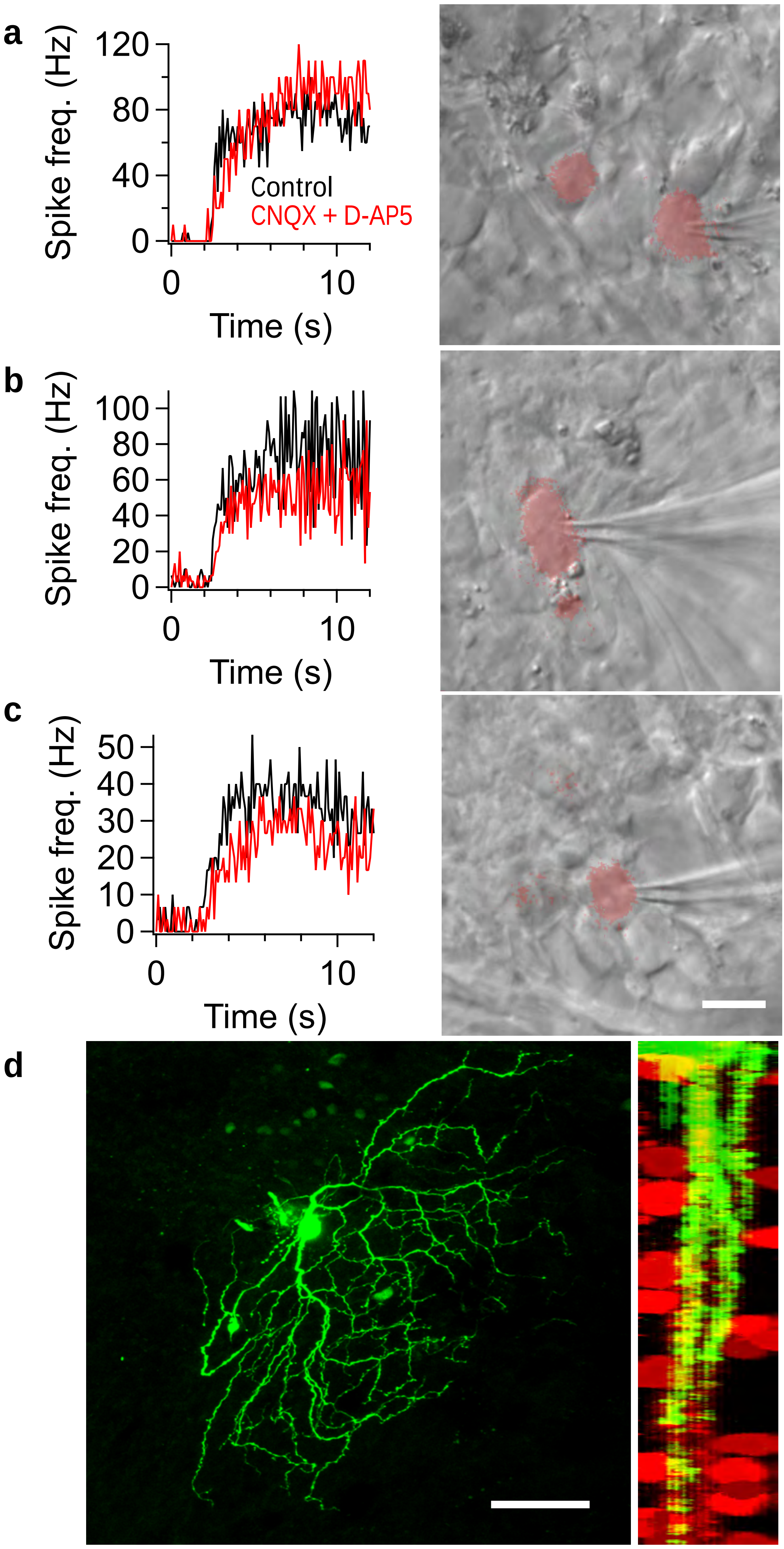

Supplementary Fig. 10: Weak off-target expression of Mela(CTmGluR6) in retinal ganglion cells makes no significant contribution to RGC response diversity. (**a-c**) In eight treated animals, we were able to record light responses from only three retinal ganglion cells (left panels) with detectable reporter gene expression (TurboFP635; right panels). Labelled RGCs were all in one piece of the same retina. Intrinsic light responses – with synaptic blockade (CNQX and D-AP5; 20μM each) – were slow and mirrored light responses of ipRGCs. (**d**) Intracellular labelling of one cell (responses shown in **c**) revealed a bi-stratified morphology and confirms that light responses were not native but introduced through ectopic expression of Mela(CT mGluR6). Scale bars = 50μm.

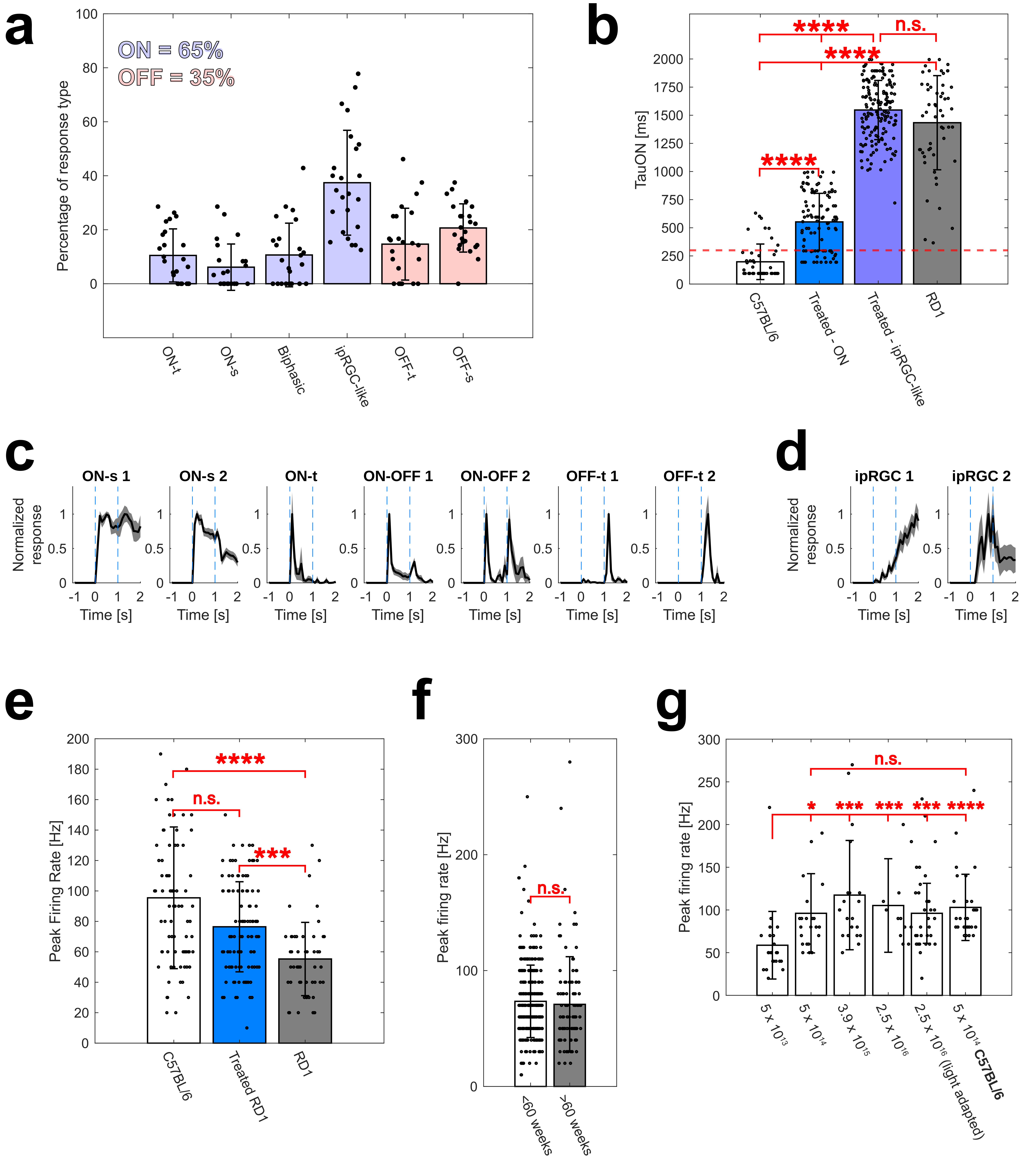

Supplementary Fig. 11: Overview of MEA recordings. (**a**) Distributions of response types in Mela(CTmGluR6) treated rd1 retinas (N=24 retinal pieces). We observed approximately 65% of ON responses (light blue; 64.68±16.18%) and 35% of OFF responses (light red; 35.32±16.18%). Dark points represent individual percentages per retinal piece. (**b**) Comparison of Tau-ONs between ON-responses in C57BL/6 animals (197.75 ±157.88 ms; n=47 cells), ON-responses derived from Mela(CTmGluR6) treated rd1 animals (552.35±253.63ms; n=107 cells), ipRGC-like responses from the same animals (1546.49±262.88; n=162 cells) and ipRGC-like responses from untreated rd1 controls (1433.63±418.29; n=53 cells). Most prominent observation was the non-significant difference between ipRGC-like responses derived from Mela(CTmGluR6) treated rd1 animals and rd1 untreated controls (p=0.0778; α=0.0083 after Bonferroni correction; 2-tailed Kolmogorov–Smirnov test). Other comparisons yielded significant differences (p=<0.000001). Dashed red line marks the time point of 300ms – 82.98% of healthy C57BL/6 and 31.78% of ON-responses derived from Mela(CTmGluR6) treated rd1 animals has its Tau-ON bellow this point. In comparison 0% of both ipRGC-like cells exhibit fast Tau-ON. (**c**) Averaged traces of different functional RGC categories in healthy C57BL/6 animals (N=6 retinal pieces; n= 84 cells) and (**d**) untreated rd1 controls (N=7 retinal pieces; n= 53 cells). Dark lines show the averaged trace, shaded area denotes SEM. Light stimulation start and end depicted as dashed blue lines. (**e**) Comparison of peak firing rates in C57BL/6 animals (95.48±46.55 Hz; n=84 cells; 1000ms light flash, 470 nm, 5 x 1014 photons/cm2/s), Mela(CTmGluR6) treated rd1 animals (76.45±29.6 Hz; n=107 cells) and untreated rd1 controls (55.28±24.07 Hz; n=53 cells). There was no significant difference between C57BL/6 animals and treated rd1 animals (p=0.0335; α=0.0166 after Bonferroni correction; 2-tailed Kolmogorov–Smirnov test), however responses observed in treated animals were significantly higher than in untreated negative controls (p=<0.00007). Only ON-responses were used. (**f**) Comparison of peak firing rates of all light responsive cells in mice aged bellow 60 weeks of age at the time of experiment (73.45±31.26; n=307 cells) and mice older than 60 weeks at the time of experiment (70.87±41.10; n=104 cells). We did not observe significant differences between these groups (p=0.117; α=0.05; 2-tailed Kolmogorov–Smirnov test). Dark spots represent individual cells. (**g**) Peak firing rates of light-responsive cells using different light intensities (n=23 cells; 470 nm; 500ms light flash). We observed significantly lower peak firing rates at 5 x 1013 photons/cm2/s in comparison to all other tested light intensities (2-tailed Kolmogorov–Smirnov test; α=0.0034 after Bonferroni correction; *=<0.0033, **=<0.000667, ***=<0.000066, ****=<0.000006). Data shown as means ± SD.

Supplementary Table 1: Overview of cell-attached patch-clamp recordings from RGCs combined with subsequent intracellular dye labelling in the treated *rd1* retina.

ID	Physiology	Anatomy	Response
1	ON	OFF	Transient
2	ON	OFF	Transient
3	OFF	OFF	Sustained
4	ON	OFF	Transient
5	OFF	OFF	Sustained
6	ON	ON	Sustained
7	ON	OFF	Transient
8	OFF	OFF	Sustained
9	ON	ON	Transient
10	ON-OFF	ON-OFF	Transient
11	ON	ON	Sustained
12	ON	OFF	Transient
13	OFF	OFF	Transient
14	ON	OFF	Transient

Comparing the light responses and corresponding anatomy of 14 dye-labelled RGCs from Mela(CTmGluR6) treated *rd1* retinas show that 60% of native OFF RGCs (n=6) have inverted light responses (red numbering; now respond at the onset of light). In the ON-type and OFF-type cells the light response did not invert. This asymmetry infers synaptic rewiring in the inner retina that differentially affect RGC subtypes after photoreceptor loss.
