# Supplementary material for: Bipolar cell targeted optogenetic gene therapy restores parallel retinal signaling and high-level vision in the degenerated retina
Source: Commun Biol. 2022 Oct 20;5:1116. doi: 10.1038/s42003-022-04016-1 (PMC9585040; doi:10.1038/s42003-022-04016-1)
Supplement: Supplementary file 3 — Description of Additional Supplementary Files [file 42003_2022_4016_MOESM3_ESM.pdf]

Description of Additional Supplementary Files

File name: Supplementary Data 1
Description: : The source data of graphs in the paper.

File name: Supplementary Movie M1
Description: Behavioural restoration of pattern recognition. Visually triggered behaviour of a Mela(CTmGluR6)-injected rd1 mouse on the evaluation day (day 4).
