# Supplementary material for: Bipolar cell targeted optogenetic gene therapy restores parallel retinal signaling and high-level vision in the degenerated retina
Source: Commun Biol. 2022 Oct 20;5:1116. doi: 10.1038/s42003-022-04016-1 (PMC9585040; doi:10.1038/s42003-022-04016-1)
Supplement: Supplementary file 6 — Reporting Summary [file 42003_2022_4016_MOESM6_ESM.pdf]

nature portfolio

Corresponding author(s): Sonja Kleinlogel

Last updated by author(s): Sep 1, 2022

Reporting Summary

Nature Portfolio wishes to improve the reproducibility of the work that we publish. This form provides structure for consistency and transparency in reporting. For further information on Nature Portfolio policies, see our Editorial Policies and the Editorial Policy Checklist.

Statistics

For all statistical analyses, confirm that the following items are present in the figure legend, table legend, main text, or Methods section.

n/a	Confirmed	
☐	☒	The exact sample size (*n*) for each experimental group/condition, given as a discrete number and unit of measurement
☐	☒	A statement on whether measurements were taken from distinct samples or whether the same sample was measured repeatedly
☐	☒	The statistical test(s) used AND whether they are one- or two-sided<br>*Only common tests should be described solely by name; describe more complex techniques in the Methods section.*
☐	☒	A description of all covariates tested
☐	☒	A description of any assumptions or corrections, such as tests of normality and adjustment for multiple comparisons
☐	☒	A full description of the statistical parameters including central tendency (e.g. means) or other basic estimates (e.g. regression coefficient) AND variation (e.g. standard deviation) or associated estimates of uncertainty (e.g. confidence intervals)
☐	☒	For null hypothesis testing, the test statistic (e.g. *F*, *t*, *r*) with confidence intervals, effect sizes, degrees of freedom and *P* value noted<br>*Give P values as exact values whenever suitable.*
☒	☐	For Bayesian analysis, information on the choice of priors and Markov chain Monte Carlo settings
☒	☐	For hierarchical and complex designs, identification of the appropriate level for tests and full reporting of outcomes
☒	☐	Estimates of effect sizes (e.g. Cohen's *d*, Pearson's *r*), indicating how they were calculated

Our web collection on statistics for biologists contains articles on many of the points above.

Software and code

Policy information about availability of computer code

Data collection	Recorded multi-electrode array (MEA) signals were collected, amplified, and digitized at 25 kHz using MCRack software (version 4.6.2, Multi Channel Systems MCS GmbH). Recordings of visually evoked potentials (VEPs) were acquired, amplified and digitized at 20 kHz with the RHD 2000 (version 1.5.2, Intan Technologies). Patch-clamp data were recorded using Patchmaster software (version 1.3, HEKA Electronics).
Data analysis	For MEA recordings, Offline Sorter (version 4.6.0, Plexon) was used for spike sorting, further analysis was done in Matlab (version R2020b, MathWorks) and GraphPad (version 9.3.1, Prism). VEPs were further analyzed using Matlab (version R2020b, MathWorks).Patch-clamp data was analyzed using Igor Pro software (version 7, WaveMetrics). Images were processed in ImageJ Fiji (version 2.1.0).

For manuscripts utilizing custom algorithms or software that are central to the research but not yet described in published literature, software must be made available to editors and reviewers. We strongly encourage code deposition in a community repository (e.g. GitHub). See the Nature Portfolio guidelines for submitting code & software for further information.

Data

Policy information about availability of data

All manuscripts must include a data availability statement. This statement should provide the following information, where applicable:

- Accession codes, unique identifiers, or web links for publicly available datasets
- A description of any restrictions on data availability
- For clinical datasets or third party data, please ensure that the statement adheres to our policy

The datasets used and/or analyzed during the current study are available from the corresponding author upon reasonable re-quest. Sequence data that support the findings of this study have been deposited in GenBank with the accession codes MQ072285.1 for Mela(CTmGluR6) and MQ072299.1 for Mela(CT+IL3mGluR6).

Field-specific reporting

Please select the one below that is the best fit for your research. If you are not sure, read the appropriate sections before making your selection.

☒ Life sciences ☐ Behavioural & social sciences ☐ Ecological, evolutionary & environmental sciences

For a reference copy of the document with all sections, see nature.com/documents/nr-reporting-summary-flat.pdf

Life sciences study design

All studies must disclose on these points even when the disclosure is negative.

Sample size	Sample sizes were mainly estimated form previous experience in experiments using similar experimental techniques.
Data exclusions	Single outlier was excluded in Figure 6A C57BL/6 as it reflected technical outlier, rather than biological phenomenon.
Replication	All data sets were presented with sample size as well as measure of variance.
Randomization	Animals were randomly allocated to treatment groups with no pre-selection criteria. We did not discriminate between male and female animals.
Blinding	Experiments were blinded and processed without human bias (e.g. automated OMR tracking and MEA cell grouping) wherever feasible.

Reporting for specific materials, systems and methods

We require information from authors about some types of materials, experimental systems and methods used in many studies. Here, indicate whether each material, system or method listed is relevant to your study. If you are not sure if a list item applies to your research, read the appropriate section before selecting a response.

Materials & experimental systems

n/a	Involved in the study	
☐	☒	Antibodies
☐	☒	Eukaryotic cell lines
☒	☐	Palaeontology and archaeology
☐	☒	Animals and other organisms
☒	☐	Human research participants
☒	☐	Clinical data
☒	☐	Dual use research of concern

Methods

n/a	Involved in the study	
☒	☐	ChIP-seq
☒	☐	Flow cytometry
☒	☐	MRI-based neuroimaging

Antibodies

Antibodies used	anti-tRFP (AB234), anti-melanopsin (AB-N39), anti-ChAT (AB144P), anti-Goα (mab3073)
Validation	All antibodies have been validated previously and labeling of target cell populations in this study were as expected and published.

Eukaryotic cell lines

Policy information about cell lines

Cell line source(s)	HEK293-GIRK cells were obtained from Prof. Olivia Masseck (University Bremen).
Authentication	Robust GPCR triggered GIRK currents confirms the description of this cell line.
Mycoplasma contamination	Routine mycoplasma tests of all our cell lines confirmed the cell line to be free of mycoplasma.
Commonly misidentified lines (See ICLAC register)	N/A

Animals and other organisms

Policy information about studies involving animals; ARRIVE guidelines recommended for reporting animal research

Laboratory animals	Mice; C3H/HeOuJ (rd1) and C57BL/6J (WT) strains; both sexes.

Wild animals	This study did not involve wild animals.
Field-collected samples	This study did not involve samples collected from the field.
Ethics oversight	Cantonal Veterinary Authority of Bern.

Note that full information on the approval of the study protocol must also be provided in the manuscript.
